# Editorial: The Emerging Role of Monocyte-Derived Cells in the Central Nervous System

**DOI:** 10.3389/fimmu.2016.00223

**Published:** 2016-06-07

**Authors:** Eva Rajnavolgyi

**Affiliations:** ^1^Department of Immunology, Faculty of Medicine, University of Debrecen, Debrecen, Hungary

**Keywords:** monocyte–macrophage precursor cells, monocyte-derived macrophages, monocyte-derived dendritic cells, neuroimmunomodulation, neuroimmune interactions

**The Editorial on the Research Topic**

**The Emerging Role of Monocyte-Derived Cells in the Central Nervous System**

Hematopoietic stem cells (HSCs) with self-renewal potential differentiate in contact with flowing blood and during embryonic development give rise to all blood cell types. Neuroinflammation is a pathological process frequently associated with tissue damage, which plays role in the clearance of pathogens and in repair mechanisms. In an original article (Wlodarczyk et al.), the authors suggest that beneficial or pathological outcomes of MS and EAE is the consequence of myeloid cell heterogeneity comprising resident microglia, infiltrating bone marrow (BM)-derived monocytes, macrophages, and dendritic cells (DCs). The CX3CR1 receptor and its ligand CX3CL1 are expressed in microglia and contribute to neuroinflammation. Disrupting this regulatory axis may result in abnormal microglial activation leading to earlier onset and higher incidence of disease manifestation. Phenotypic and functional analyses revealed that CD11c(+) and CD11c(−) microglia and CD11c(+) blood cells represent functionally different populations exhibiting pathological and protective functions in line with inflammatory gene expression profiles. At the peak of EAE, a subpopulation of microglia expresses IFNβ that confers protection verified by upregulated CSF-1 production.

Dendritic cells represent a diverse population of cells, which continuously sense and digest soluble/particulate antigens and their migratory potential helps to eliminate harmful tissue/cell components. Recent data revealed that microglia acquires a dysfunctional rather an activated phenotype causing defective clearance of harmful structures together with the loss of neuronal support, whereas systemic inflammation causes the exacerbation of neurodegenerative diseases. The perspective article by Bossù et al. focuses on the collaboration of the immune and the nervous systems with emphasis on the elimination of harmful material linked to inflammation and neuronal loss. These processes also disturb the blood–brain barrier leading to vascular dysfunction through peripheral cells. Recent evidence suggests that blood monocytes are associated with familiar and sporadic forms of AD and PD, and CD33 and TREM2 gene variants have been identified as risk factors of AD associated with PD and coupled with myeloid cells, which infiltrate the degenerated brain, while the clearance of vascular Aβ is carried out by patrolling monocytes. According to a recent report by Louveau et al. (1) found that the CNS is monitored by unique immune surveillance mechanisms showing that the trafficking of leukocytes into the CNS through lymphatic vessels provides a novel route for the entrance/exit of various cell types, among them DC subsets supporting regulatory events. State-of-the-art techniques may help to identify DC functions at levels of initiation and/or regulation during brain-specific immune responses. Potential targets for therapeutic approaches may rely on DC-based vaccination strategies rather than modifying ongoing immune responses. Optimized immunization protocols to eliminate accumulated misfolded proteins may offer novel means to decrease pathological consequences in the CNS. In this context, Ab-sensitized DCs have successfully been applied to reduce Aβ accumulation and attenuate cognitive deficit.

Circulating extracellular macrovesicles (EVs) have been shown to contribute many disorders and elevated numbers of shedding vesicles in plasma and/or in body fluids have also been identified. Importantly, the appearance of these vesicles correlates with acute or active phase of diseases and can be used as predictive and/or diagnostic biomarkers for pathological conditions. As a novel aspect, the article by Nigro et al. addressed the question how intracellular vesicles could modify the outcome of MS at the progressive phase of the disease and in classical neurodegenerative conditions including AD and PD. The molecular composition of shedding vesicles and exosomes was found to be heterogeneous and contain signaling molecules, receptors, integrins, cytokines, bioactive lipids, nucleic acids, and organelles thus acquiring the potential to modify the activities of the recipient cells. Despite considerable heterogeneity of exosomes, their cellular origin is shared. This study also demonstrates that the increase of EVs, derived from myeloid cells of AD patients and with mild cognitive impairment, correlates with classical biomarkers of neuronal injury demonstrating the diagnostic potential of EVs. When microglia-derived EVs were exposed to the amyloid-β^1–42^ peptide, soluble neurotoxic species derived from extracellular insoluble aggregates were generated, which exhibited potent neurotoxicity. This finding, in line with the observation that activated microglia surround amyloid deposits, suggests the involvement of cell-to-cell communication supporting the spread of amyloid-β^1–42^ pathology throughout the brain.

The etiology of MS has been associated with both genetic predisposition and environmental factors, including exogenous retroviruses, which have acquired the capability to get integrated into human germ line cells and transmit them over generations through Mendelian trait. MS is associated with HERV-H, K, W retroviruses commonly referred to as MSRV. The article by Morandi et al. suggests that HERVs act as pathogenic factors linking the individuals’ genetic susceptibility to CNS autoimmunity and infections acting as disease triggers. In most cases, although retroviruses are silenced or expressed at low levels, they may increase expression. HERVs are expressed in monocytes, microglia, and within CNS lesions of MS patients, and upon TLR4 receptor-mediated activation they produce pro-inflammatory cytokines, reduce myelin expression, and cause death of oligodendrocyte precursors. MS is the most common cause of neurological disability in young adults manifested as a chronic demyelinating process in the CNS with inflammatory and degenerative changes in the brain and spinal cord. During MS, high level of HERV-W/MSRV promotes disease progression increasing with time of relapses confirming its association with HERV-W/MSRV.

Epigenetic states are defined at the level of DNA and chromatin modifications and by RNA editing processes. The state-of-the-art review by Papavasiliou et al. discusses the advantage of newly developed technologies that allow capturing the epigenetic status of individual genes in a time-dependent manner thus providing information on transcriptional responses during development and in response to environmental stimuli. The authors of this article discuss how epigenetic modulations may affect the development of a healthy brain as opposed to neurodegenerative diseases and aging. For example, DNA methylation is an epigenetic modification, which regulates X chromosome inactivation, cell fate, chromosomal instability, repression of transposable elements, and gene expression during lifetime of the organism. The level of CpG methylation inversely correlates with gene expression and controls transcription either locally or through occluding transcription factor binding sites within promoter regions. This is accomplished *via* binding DNA and interfering with transcriptional elongation, or globally through methylation of CpG-rich regions leading to the recruitment of proteins that alter the chromatin state and result in general locus silencing. Covalent modifications impact gene expression levels and respond to environmental stimuli such as migration of cells to the site of infection or into lymph nodes guided by the chemokine CCL2. According to a recent report, this process is supported by the coactivator protein IκBz supporting the action of CCL2 (2). In conclusion, the mechanisms outlined in this issue demonstrate the emerging importance of future investigations into the very nature of neuroimmune communication. Targeting monocyte-derived cells in the CNS offers a promising and novel approach for designing immunomodulatory strategies that may offer potent treatment options for neuro- and psychiatric diseases.

## Author Contributions

The author confirms being the sole contributor of this work and approved it for publication.

## Conflict of Interest Statement

The author declares that the research was conducted in the absence of any commercial or financial relationships that could be construed as a potential conflict of interest.
